# Transcatheter closure for patent ductus arteriosus in patients with Eisenmenger syndrome: to do or not?

**DOI:** 10.1186/s12872-020-01795-5

**Published:** 2020-12-01

**Authors:** Jing Xu, Liang Wang, Yunli Shen, Liang Geng, Fadong Chen

**Affiliations:** Department of Cardiology, Shanghai East Hospital, Shanghai Tongji University School of Medicine, No. 150, Jimo Road, Pudong District, Shanghai, 200120 China

**Keywords:** Patent ductus arteriosus, Eisenmenger syndrome, Transcatheter closure, Diagnostic treatment and repair strategy, Targeted drugs

## Abstract

**Background:**

Patent ductus arteriosus (PDA) complicated by Eisenmenger syndrome (ES) remains to be a major cause of morbidity and mortality worldwide. Giving increasing evidences of benefit from targeted therapies, ES patients once thought to be inoperable may have increasing options for management. This study aims to explore whether PDA in patients with ES can be treated with transcatheter closure (TCC).

**Methods:**

Between August 2014 and July 2016, four of fifteen PDA-ES patients whose Qp/Qs improved significantly and Qp/Qs > 1.5 after acute vasodilator testing with 100% oxygen were selected to receive TCC and pulmonary vasodilator therapy. PAH-targeted drugs were prescribed before and after occlusion for all. Trial occlusion was performed before permanent closure.

**Results:**

The first TCC failed after initiation of PAH-targeted drugs for 6 months in four patients. After the medication was adjusted and extended to 12 months, TCC was performed for all without hemodynamic intolerances during perioperative period. Pulmonary artery systolic pressure (PASP) was significantly decreased (≥ 40%) immediately after TCC. During a mean follow-up of 48 ± 14.70 months, there were a further decrease of PASPs in two patients, the other two showed improved pulmonary vascular resistance, WHO functional class and six-minute walking distance despite deteriorated PASP.

**Conclusion:**

Some selected PDA-ES patients might benefit from TCC and combined PAH-targeted drugs play a crucial role.

## Background

Patent ductus arteriosus (PDA) is one of the most common congenital heart defects (CHDs). Without timely correction, vasomotor dysfunction of endothelial cells and vascular remodeling will develop gradually in pulmonary arteries, leading to increased pulmonary vascular resistance (PVR), severe pulmonary arterial hypertension (PAH) and eventually Eisenmenger syndrome (ES) which remains to be a major cause of morbidity and mortality worldwide [1, 2]. Additionally, in developing countries such as China, PDA associated with ES is common because CHDs are not detectable until adulthood and thereby ES has developed. This situation is now becoming a frontier issue.

Transcatheter closure (TCC) for PDA has been established as a safe and effective alternative to surgical closure with the advancement and improvement of techniques and materials [3]. However, TCC is generally considered as contraindicated for ES patients due to irreversible obstructive lesions of the pulmonary vasculature in the past clinical practice.

Recently, giving increasing evidence of benefit from targeted therapies [4], ES patients once thought to be inoperable may have increasing options for management [5, 6]. Patients with severe PAH are amenable to receive surgery or TCC after successful treatment with targeted drugs [7–9]. However, the immediate and long-term prognosis with such patients is unknown.

In this study, we aim to study the change of pulmonary artery systolic pressure (PASP), cardiac function and hemodynamic variables of four PDA-ES patients who underwent TCC and pulmonary vasodilator therapy by diagnostic treatment and repair strategy with long-term follow-up, in order to identify whether PDA-ES patients can benefit from TCC.

## Methods

### Patients

The records of fifteen patients with clinical and echocardiographic findings of PDA and ES were retrospectively reviewed from August 2014 to July 2016. The inclusion criteria for ES are based on European guidelines [10]. Each patient was evaluated by arterial blood gas analysis, six-minute walking distance (6MWD), World Health Organization functional class (WHO FC), echocardiography and finally right heart catheterization (RHC). This study was conducted in accordance with the amended Declaration of Helsinki. Written informed consents were obtained from all the patients.

### Hemodynamic measurement

RHC was performed with Swan-Ganz catheter (Edwards 774,7.5F) and monitoring system (Edwards Lifesciences LLC, Vigilance II). All measurements were performed in supine position. Hemodynamic parameters included right atrial pressure (RAP), pulmonary artery pressure (PAP) and pulmonary artery wedge pressure (PAWP). Cardiac output (CO) were assessed using the Fick’s method before TCC or continuous thermodilution method during follow-up. Arterial blood gases and mixed venous oxygen generation (SvO_2_) were also measured. Pulmonary to systemic flow ratio (Qp/Qs), PVR and systemic vascular resistance (SVR) were calculated using standard formulas. All measurements were made in a stable baseline condition without oxygen for 2 h at least.

Acute vasodilator testing was then performed with oxygen. Standardized oxygen was provided via standard commercial equipment at a flow rate of 8 L/min, achieving an oxygen saturation of 100% in every patient. Oxygen was applied at least 10 min. Hemodynamic parameters, particularly Qp/Qs, were again recorded. Qp/Qs > 1.5 after inhalation of 100% oxygen was defined as an absolute cutoff value to screen the candidates for our study.

### Intervention procedure

Under local anesthesia and transthoracic echocardiographic guidance, interventional procedure was performed after percutaneous puncture of the femoral artery and vein. Morphology of PDA was demonstrated by descending aorta angiography with 6F pigtail catheter, and the narrowest diameter of PDA was measured meanwhile. Trial occlusion using PDA occluder(Shanghai Shape Memory Alloy Ltd, China) was performed for 30 min to measure the change of hemodynamic data. The occluder was released when all the following criteria were satisfied after trial occlusion: (1) a decrease in PASP ≥ 40%; (2) no decrease in aortic pressure (AOP); (3) an increase in systemic arterial oxygen saturation (SaO_2_).

### Follow-up

The patients were followed up in out-patient clinic every 6 months after discharge with the last follow-up in August 2020. 6MWD, transthoracic echocardiography and blood gas analysis were routinely carried out. The hemodynamic evaluations by RHC were assessed in case 2,3,4 at 72,48,36 months follow-up, respectively.

## Results

### Study patients

**Fig.1:**
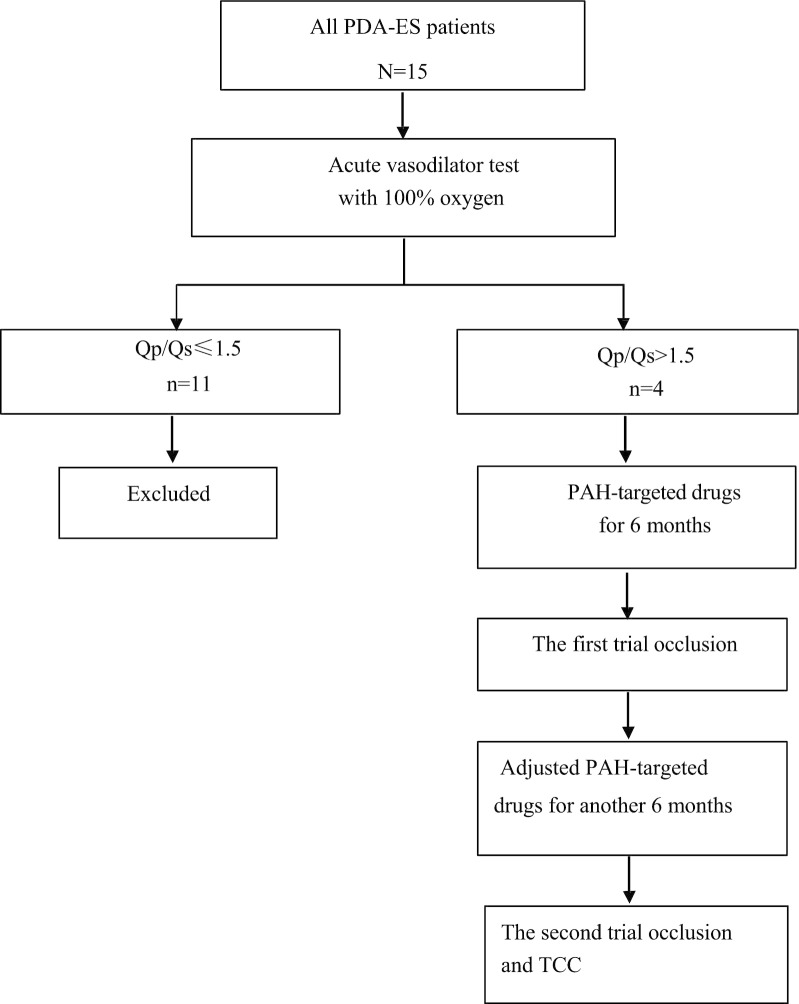
Study flow chart. *PDA* patent ductus arteriosus, *ES* Eisenmenger syndrome, *Qp/Qs* pulmonary-systemic blood flow ratio, *PAH* pulmonary arterial hypertension, *TCC* transcatheter closure

After diagnosis by initial RHC and acute vasodilator testing, four (3 female and 1 male) of fifteen PDA-ES patients were finally selected to be treated with PAH-targeted drugs and subsequent TCC (Fig. 1).

The mean age of the four PDA-ES patients were 28.5 years (ranging from 19 to 34 years) with WHO FCII-III. Baseline demographic characteristics and echocardiography parameters were shown in Table 1.

**Table 1:** Baseline demographic characteristics and echocardiography parameters of all patients

Patient (no.)	Sex	Age (years)	WHO FC	6MWD (m)	LVEF (%)	RV diameter (mm)	PASP (mmHg)
1	M	29	III	440	53	69 * 42	115
2	F	19	II	400	67	57 * 29	120
3	F	32	II–III	170	72	68 * 40	144
4	F	34	III	450	67	75 * 35	104

*WHO FC* WHO functional class, *6MWD* six-minute walking distances, *PASP* pulmonary artery systolic pressure, *LVEF* left ventricular ejection fraction, *RV* right ventricle

The mean PVR was 22.19 Wood U (ranging from 14.70 to 36.91 Wood U). The mean PASP and AOP were 126 mmHg (ranging from 105 to 145 mmHg) and 129 mmHg (ranging from 113 to 144 mmHg), respectively. Baseline hemodynamic parameters obtained by RHC and the changes of Qp/Qs after 100% oxygen inhalation were shown in Table 2.

**Table 2:** Baseline hemodynamics parameters measured by right heart catheterization

Patient (no.)	PDA-SD	RAP (mm Hg)	PA (mm Hg)			AO (mm Hg)			TPR (Wood U)	PVR (Wood U)	SVR (Wood U)	PVR/SVR	SaO_2_ (%)	Qp/Qs	
			SP	DP	MP	SP	DP	MP						Baseline	O_2_ test
1	Bidi	13/6/9	105	73	81	114	69	79	18.95	16.14	28.29	0.57	91.8	1.73	2.92
2	Bidi	5/0/2	105	65	85	115	65	80	15.82	14.70	40.34	0.36	95	2.13	2.46
3	Bidi	9/2/5	143	63	95	142	62	95	40.31	36.91	36.07	1.02	88.9	1.00	1.72
4	Bidi	11/3/7	134	72	98	130	70	90	23.13	21.01	24.50	0.86	89.7	1.10	2.60

*PDA-SD* shunt direction across PDA, *Bidi* Bi-directional shunt; RAP, right atrium pressure, *PA* pulmonary artery, *AO* aortic pressure, *SP* systolic pressure, *DP* diastolic pressure, *MP* mean pressure, *TPR* total pulmonary resistance, *PVR* pulmonary vascular resistance, *Qp/Qs* pulmonary-systemic blood flow ratio, *SVR* systemic vascular resistance, *SaO*_*2*_ systemic arterial oxygen saturation

### Diagnostic treatment and repair strategy

After initiation of PAH-targeted therapy for 6 months, the first TCC attempt failed because PASPs of the four patients during trial occlusion did not decrease or the reduction was less than 20%. After targeted therapy was adjusted and extended to 12 months, all the criteria were met and the PDA occluder was released following trial occlusion. There was no residual shunt for all and no complication or adverse event during perioperative period. All patients were discharged with PAH-targeted drugs 1–2 days after TCC. Initial and adjusted PAH-targeted drugs before permanent TCC were shown in Table 3. The PDA diameter, occluder size, changes of PASP, AOP and SaO_2_ before and after trial occlusion were shown in Table 4.

**Table 3:** Initial and adjusted PAH-targeted drugs before TCC

Paitent (no.)	Intial	Adjusted
1	Vardenafil 5 mg bid	Vardenafil 5 mg bid Bosentan 125 mg bid
2	Tadanafil 20 mg qd	Tadalafil 20 mg qd Bosentan 125 mg bid
3	Bosentan 125 mg bid Tadanafil 20 mg qd	Bosentan 125 mg bid Tadanafil 20 mg qd
4	Ambrisentan 5 mg qd Tadalafil 20 mg qd	Ambrisentan 5 mg qd Tadalafil 20 mg qd

**Table 4:** Comparisons between pre- and post-occlusion parameters

Patient (no.)	PDA diameter (mm)	Occluder size (mm)	PA (mmHg)						AO (mmHg)						SaO_2_ (%)	
			Pre-			Post-			Pre-			Post-			pre-	post-
			SP	DP	MP	SP	DP	MP	SP	DP	MP	SP	DP	MP		
1	10	18–20	105	54	72	56	34	43	113	73	85	124	86	108	96	98
2	9	16–18	110	61	84	61	44	53	123	69	94	116	69	91	97	100
3	9	20–22	138	58	88	74	27	46	137	67	89	150	70	100	92	100
4	11	20–22	145	72	104	72	30	48	144	78	102	153	91	116	97	100

*PDA* patent ductus arteriosus, *PA* pulmonary artery, *AO* aorta, *SP* systolic pressure, *DP* diastolic pressure, *MP* mean pressure, *SaO*_*2*_ systemic arterial oxygen saturation

### Follow-up

**Fig.2:**
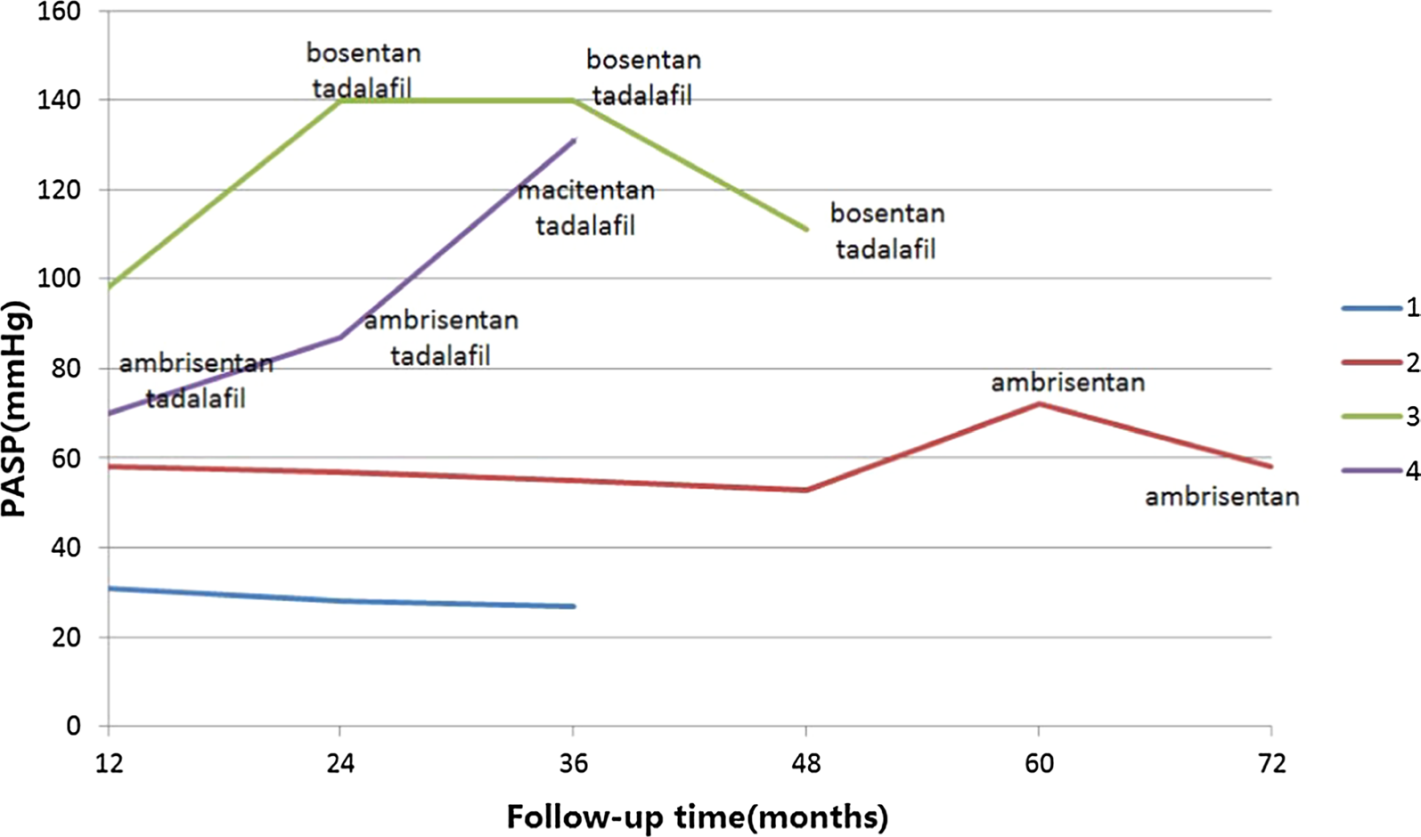
PASP changes on echocardiography and adjustment of targeted drugs at follow-up after final TCC. *PASP* pulmonary artery systolic pressure, *TCC* transcatheter closure

At 12-month follow-up, Cases 1 and 2 discontinued targeted therapy because PASP decreased to near normal. Case 2 was treated with ambrisentan again at 60-month as PASP rose to 72 mmHg. At 72-month follow-up, the PASP fall to 58 mmHg.PASP of Case 3 decreased to 98 mmHg at 12-month but rose to 140 mmHg at 24-month follow-up after stopping targeted drug without doctor consultant, therefore she was prescribed with bosentan and tadanafil again and the PASP was 111 mmHg at 48-month follow-up. PASP of Case 4 decreased to 70 mmHg at 12-month, but the PASP rose again to 87 mmHg and 131 mmHg at 24-month and 36-month, respectively. She was prescribed with macitentan instead of ambrisentan at 29-month follow-up. The changes of PASP measured by echocardiography and targeted drugs adjustment of each individual during follow-ups after final TCC were shown in Fig. 2.

All the four patients showed improved 6MWD, WHO FC and SaO_2_ without enlarged RV diameter during a mean follow-up of 48 ± 14.70 months (ranging from 36 to 72 months). Hemodynamic assessment by RHC showed there was a significant fall in PVR of 3.83Wood U in case 2. Case 3 and 4 also displayed improved PVR of 12.68Wood U,12.54 Wood U and PVR/SVR of 0.88,0.80, respectively (Table 5). RV diameter, WHO FC, 6MWD and PAH-targeted drugs at the last follow-up were shown in Table 6.

**Table 5:** Hemodynamics parameters measured by right heart catheterization at follow-up

Paitent (no.)	RAP (mmHg)	PASP (mmHg)	CO (L/min)	SaO_2_ (%)	PVR (Wood U)	SVR (Wood U)	PVR/SVR
2	11/6/9	55/18/36	6.0	98	3.83	13.33	0.29
3	15/8/11	139/47/82	5.6	94.3	12.68	14.29	0.88
4	12/1/6	136/54/86	5.9	92.2	12.54	15.67	0.80

*RAP* right atrium pressure, *PASP* pulmonary artery systolic pressure, *CO* cardiac output, *SaO*_*2*_ systemic arterial oxygen saturation, *PVR* pulmonary vascular resistance, *SVR* systemic vascular resistance

**Table 6:** RV diameter, WHO FC,6MWD and PAH-targeted drugs at the last follow-up

Paitent (no.)	RV diameter (mm)	WHO FC	6MWD (m)	PAH-targeted drugs
1	50 * 25	I	550	–
2	55 * 28	I	500	Ambrisentan
3	65 * 40	II	440	Bosentan, tadalafil
4	59 * 39	II	490	Macitentan,tadalafil

*RV* right ventricle, *WHO FC* WHO functional class, *6MWD* six-minute walking distances

## Discussion

CHDs patients with ES were previously considered to have irreversible pulmonary hypertension. Isolated correction of the cardiac defect in patients with ES has typically been considered as contraindication [11]. Historically, management options for patients with ES have been limited to palliative measures or heart–lung transplantation. The recent introduction of targeted therapies in PAH has led to a renewed insight in the pathophysiology and treatment of ES [11, 12]. Considering ES patients maintain some degree of pulmonary vasoreactivity despite the presence of obstructive pulmonary hypertension [13], ES patients using a diagnostic treatment and repair strategy are amenable to receive surgery or TCC after successful treatment with advanced therapy, but no proof of its efficacy has really been shown in large-scale studies [14–16]. Our study indicated that some selected PDA-ES patients might be amenable to and benefit from TCC over a long follow-up period. Uninterrupted combination of PAH-targeted drugs before and after occlusion play a crucial role especially for the high-risk PDA-ES patients.

It is strongly recommended that PAH-targeted therapies [17–19] for a sufficient period of time to assess the hemodynamic and symptomatic response before closure [13, 14]. Supomo et al. [20] described a atrial septal defect(ASD)-ES female with highly symptomatic PAH(NYHA class III, mean PAP 77 mmHg, PVR 4 Wood U) underwent occlusion successfully after oral beraprost for 2 years. After surgery her mean PAP decrease to 38 mmHg with PVR of 2.52 Wood U. Hu et al. [21] reported a ventricular septal defect (VSD)-ES patient with initial PVR of 18.84 Wood U underwent a successful operation after oral bosentan treatment for 12 weeks, as a result of which her PVR decreased to 9.63 Wood U. Four PDA patients in our study were all ES with higher PASP and PVR, indicating that initial combination of PAH-targeted drugs for 1 year at least may provide ES patients a better occlusion opportunity. Especially for ES patients as case 3 and 4 with baseline PVR > 15Wood U and Qp/Qs < 1.5, initial dual or triple combination of PAH-target drugs for a longer period of time before occlusion are needed to be taken into account.

Selecting ES patients who can be treated with TCC is an important issue that needs to be addressed. The correction indications for PDA patients with severe PAH are not uniformly defined, including pulmonary artery vasoreactivity and/or the presence of Qp/Qs at least 1.5 to 1.0 [2, 10]. A strength of the this study is that all of our patients were classified as ES according to the recent definition [10], the Qp/Qs of the four patients improved significantly and Qp/Qs > 1.5 following pulmonary vasoreactivity testing were identified with preserved pulmonary vasodilation, which may be deemed candidates for pulmonary vasodilator therapy and TCC.Of note, after PAH-targeted therapy, significant fall of PASP during trial occlusion indicates a likelihood for final TCC [22, 23]. Yan et al. [24] reported successful occlusion in twenty PDA patients with mean PASP 104 mmHg, PVR 9.1 Wood U and Qp/Qs 2.1. A decrease of > 25% in PASP following trial occlusion was used as the criterion for occlusion. Thanopoulos et al. [25] reported a decrease of > 30% in PASP as occlusion criterion in seven PDA patients with Qp/Qs ≥ 2.0. Considering our four patients were all ES patients with higher PASP and lower Qp/Qs, our occlusion criteria were stricter. TCC was performed if all the following criteria were met: (1) a drop of ≥ 40% in PASP; (2) no decrease in AOP; (3) an increase in SaO_2_. During the follow-up, PASP of case 1,2 decreased further while the other two rose again, therefore the most optimal occlusion criteria were still needed further explored in a larger sample size.

During the long-term follow-up, our four patients displayed improved WHO FC and 6MWD. The PASP of Case 2 deteriorated after interruption of targeted therapy. However, the PASP improved gradually after receiving PAH-targeted therapy again, indicating the PAH of Case 2 was partially reversible thus targeted therapy could not be discontinued after TCC. Monotherapy would be adoptable needing to be maintained for a long or life-long period. The deteriorated PASPs of Case 3 and 4 indicated the two patients had inadequate PAH-targeted therapy or they might have irreversible pulmonary vascular lesions, therefore more aggressive targeted therapy is needed before and after intervention. Our result suggested uninterrupted dual or triple combination of targeted drugs including oral and intravenous or subcutaneous prostacyclin analogues are also considered for these high-risk PDA-ES patients after TCC. It is worth noting that PVRs of Cases 3 and 4 decreasing at follow-up in our study suggested improvement of right cardiac function after TCC and pulmonary vasodilator therapy. In general, in spite of initial positive pulmonary vasoreactivity testing and improved hemodynamic status after PAH-targeted therapy, TCC should be performed with caution for such special individuals.

### Study limitations

There are three main limitations in our study. First, the major limitation of the study was the small sample which limited its power. Second, PASP was evaluated by transthoracic echocardiography rather than RHC in most follow-up time. Third, standard pulmonary vasoreactivity testing should include inhaled nitric oxide in addition to 100% oxygen whereas nitric oxide was not used in our study.

## Conclusion

PDA-ES patients whose PVR ≥ 15Wood U at baseline and Qp/Qs > 1.5 on oxygen study might be amenable to and benefit from TCC, after extended period of targeted pulmonary vasodilator therapy up to 1 year. Uninterrupted dual or triple combination of PAH-targeted drugs pre-and post-occlusion play a vital role especially for the high-risk PDA-ES patients.

## Data Availability

The datasets used in the case are available from the corresponding author upon reasonable request.

## Abbreviations

PDA: Patent ductus arteriosus
ES: Eisenmenger syndrome
TCC: Transcatheter closure
PASP: Pulmonary artery systolic pressure
CHDs: Congenital heart defects
PVR: Pulmonary vascular resistance
PAH: Pulmonary arterial hypertension
6MWD: Six-minute walking distance
WHO FC: World Health Organization functional class
RHC: Right heart catheterization
RAP: Right atrial pressure
PAP: Pulmonary artery pressure
PAWP: Pulmonary artery wedge pressure
CO: Cardiac output
SvO_2_: Mixed venous oxygen generation
Qp/Qs: Pulmonary to systemic flow ratio
PVR: Pulmonary vascular resistance
SVR: Systemic vascular resistance
AOP: Aortic pressure
SaO_2_: Systemic arterial oxygen saturation
O_2_: Oxygen
